# The evolution and adoption of World Health Organization policy guidelines on antiretroviral therapy initiation in sub-Saharan Africa: A scoping review

**DOI:** 10.4102/sajhivmed.v21i1.1103

**Published:** 2020-09-30

**Authors:** Sabina M. Govere, Moses J. Chimbari

**Affiliations:** 1 College of Health Sciences, University of KwaZulu-Natal, Durban, South Africa

**Keywords:** ART initiation, WHO-ART guidelines adoption, implementation of ART guidelines in sub-Saharan Africa, CD4, human immunodeficiency virus

## Abstract

**Background:**

Despite past and present global interventions, the human immunodeficiency virus (HIV) pandemic remains a public health problem in low- and middle-income countries (LMICs). The World Health Organization (WHO) has assisted these countries by providing antiretroviral therapy (ART) policies for adoption and adaptation to local needs.

**Objectives:**

This article describes the response of countries in sub-Saharan Africa (SSA), to the WHO’s changing CD4-threshold ART-initiation recommendations of the past two decades.

**Methods:**

Relevant articles published in international peer-reviewed journals were accessed via the following search engines: PubMed, Google Scholar, Cochrane, Embase and EBSCOhost. The study’s inclusion criteria were articles published in the English language between 2000 and 2019 that highlighted changes to the CD4 ART-initiation threshold and that focused on the WHO’s ‘commencement of ART’ policy guidelines. Sixteen studies (*n* = 16) from SSA were identified and included in this review: four are cross-sectional, four deal with cost-effectiveness, four are retrospective, one is a randomised trial and three are observational studies. Only studies conducted in SSA were assessed.

**Results:**

Four themes emerged: (1) adoption of the WHO CD4-ART-initiation policy by SSA countries, (2) timely implementation of the changing guideline initiation policy in the region, (3) barriers and facilitators encountered in the implementation of the changing guidelines and (4) description of similarities in policy implementation at country level from 2002 to 2019. Regional studies – cross-sectional, observational, retrospective, cost-effectiveness and randomised have described greater access to ART in SSA. However, barriers remain. The most common barriers to the timely implementation of ‘new’ ART-initiation guidelines were economic constraints, drug stock-outs, delays in obtaining baseline blood-test results and staff shortages.

**Conclusion:**

Although countries in SSA have adopted the WHO-ART-CD4 initiation-threshold policy guidelines, implementation has seldom occurred in a timely manner. Barriers have been identified. Whilst a small number of countries have implemented recommendations promptly, for many, the barriers still require to be overcome.

## Background

The first cases of the acquired immunodeficiency syndrome (AIDS) were reported in 1981. Since then, infection with human immunodeficiency virus (HIV) has spread globally and caused an estimated 74.9 million infections and 32 million AIDS-related illnesses.^1^ In its first 15 years no treatment could control the infection or halt its spread.^2^ By 2018, the African region was home to approximately 25.7 million people living with HIV (PLWH)^1^ and in that year alone, Africa experienced approximately 1.1 million new infections.^1^ Almost two-thirds of all new global infections occur in sub-Saharan Africa (SSA).^1^

The World Health Organization’s (WHO’s) antiretroviral therapy (ART) initiation-guidelines have changed substantially over the last two decades.^2^ The guidelines were first published in 2002.^3^ These (2002/2003) recommended starting ART in those with AIDS-related conditions and/or at a CD4 of ≤ 200 cells/mm. The available treatment at that time was expensive and toxic. Delaying ART until the CD4 reached levels < 200 c/mm^3^ was intended to minimise these drawbacks.^4^ Continued deaths from AIDS and success with ART prompted a CD4 increase in 2006 200 to 350 cells/mm^3^. In addition, all pregnant women and persons with Stage 3 and 4 infection were offered ART.^3^ In 2010, the threshold was raised to CD4 < 350 c/mm^3^ for all irrespective of clinical stage.^4,5^ By June 2013, the threshold was further increased to CD4 < 500/cells/mm^3^ for all children > 5 years and adults irrespective of stage/symptoms.^6^ In 2015, the WHO and numerous international organisations removed the CD4 threshold and recommended ART to all regardless of CD4 cell count and clinical stage.^7^ Data from two highly influential randomised controlled clinical trials, the START and TEMPRANO studies, underpinned this decision. Both demonstrated survival advantage to those on ART irrespective of clinical stage or CD4 count.^8,9^ This led to the introduction by all international agencies, including the WHO, of the policy of ‘universal test and treat (UTT)’. The WHO estimates that if these recommendations are adopted globally, 21 million deaths and 28 million new infections could be prevented by 2030.^10^

The rate at which countries have aligned their national ART programmes and implemented WHO guidelines since 2002 has varied. Most SSA countries took ± 2 years to implement the WHO’s 2010 ART guidelines.^5^ From December 2015 to May 2017, Rwanda, Kenya, Uganda, Botswana, Malawi, Zimbabwe and South Africa revised national ART eligibility guidelines to align with the WHO’s 2015 guidelines.^11^ On average, this integration took 12 months (range, 6–23 months).^11^ The implementation of the WHO guidelines in resource-constrained countries is complex. Consequently, it has not always been possible to implement the guidelines timeously where ART is most needed and where access to health services is limited.^2^ In this review, we sought to determine how different SSA countries adapted to the WHO’s ART-initiating CD4-threshold changes over time and how WHO guidelines have impacted ART in the region.

## Methods

### Search strategy and selection criteria

**FIGURE 1:**
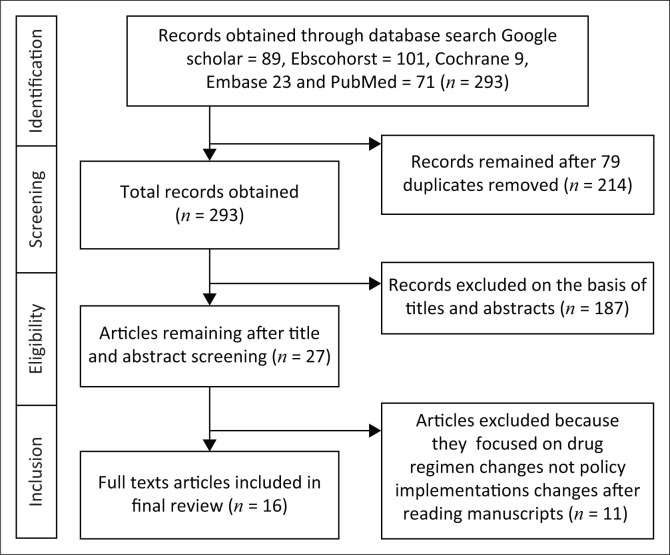
Preferred reporting items for systematic reviews and meta-analysis flow diagram showing the process of selecting articles included in the review.

We carried out a systematic electronic literature search on PubMed, Google Scholar, Cochrane, Embase and EBSCO host for the period, 2000–2019 (Figure 1). The databases were selected based on our inclusion criteria and the availability of free full-text articles and papers. In this review, we used the preferred reporting items for systematic reviews and meta-analysis (PRISMA) as described by Moher et al., to identify an evidence-based dataset and to provide transparency in the selection process of the articles.^12^

The search was based on the combination of the following terms and Boolean operators: WHO-ART guidelines or ART-initiation guidelines and changes in CD4-initiation guidelines and implementation of WHO guidelines or adoption of WHO-ART guidelines. We also applied a manual country filter to limit our search to SSA. Articles published in a language other than English and articles focusing on ART regimen-change were excluded. The study included articles that focused on CD4-threshold changes and were published between 2000 and 2019. The following articles were not included: duplicates, articles not centered on the WHO and ART initiation guidelines or their adoption and implementation. Exclusion was based on the screening of the title and abstract.

The search process is illustrated in Figure 1. Seventy-nine (79) duplicate articles were removed, which were identical in Google Scholar and PubMed. Fewer articles dealt with the topic in Cochrane and Embase. The articles in PubMed were more detailed, easier to search and free to access. We also excluded 187 articles because they did not specifically address implementation based on CD4-threshold changes. Another 11 were excluded because they focused on only regimen change. Only 16 articles remained. These covered quantitative and qualitative synthesis of how SSA countries adopted the WHO and ART initiation guidelines between 2000 and 2019 and its impact on the management of HIV.

### Data extraction and synthesis

The following information was extracted from selected studies using a template: publication details, country of study, objective(s) of the study, study design, summary of findings and theme (Table 1). Two review authors independently assessed the eligibility of the studies identified in the search. Articles with different study designs and objectives were selected to reduce the risk of bias. We used different high-impact databases to search for articles and global authors. The study designs were divided into five groups: cross sectional, cost-effectiveness, retrospective, randomised trial and observational studies. We did not subject the reviewed articles to this quality process because this is a scoping review. For synthesis, extracted information was grouped into themes derived from the articles in line with the review objectives and different study designs. The themes identified were: how different SSA countries adopted WHO and ART initiation policy guidelines at country level, timely implementation levels of the policies by different SSA countries, the barriers and facilitators to WHO and ART initiation policy adoption in SSA and the similarities in country characteristics in policy implementation in different SSA countries.

**TABLE 1:** Summary of studies.

Author and year	Guidelines year	Theme identified	Study objectives	Type of study	Study focus	Study location	Major outcomes of study	Strength/weakness of design
Ambia et al.^13^ 2017 Paper 1	2013	Timely implementation of WHO and ART initiation policy guidelines at country level. Adoption of WHO and ART initiation policy guidelines at country level Barriers and facilitators to WHO-policy implementation	The study assessed the uptake of the 2013 WHO recommendations related to the eligibility threshold for ART-initiation, the availability of first-line ART- regimens, and recommendations to improve retention.	Cross-sectional survey	Inclusion of the 2013 WHO HIV treatment recommendations	Kenya, Malawi, South Africa, Tanzania, Uganda and Zimbabwe	Although expansion of ART access was explicitly stated in all countries’ policies, most lacked policies that enhanced retention. The proportion of facilities that initiated ART at CD4 counts of 500 or less cells/mm^3^ increased from 12% to 68%. Treatment stock-outs affected increase in ART enrolment. Facilities initiating patients onto 2013 WHO recommended ART-regimen increased from 42% to 87%.	To their knowledge this was the first study to use two sequential cross-sectional surveys to compare implementation of policies on ART access and retention across six African countries with a generalised HIV epidemic. The conceptual framework that underpinned this study was developed prior to the first facility survey round and was based on a review of literature and policy in circulation up to 2015. Whilst comprehensive at the time, the provision of HIV care and treatment is a rapidly evolving field and it is possible that additional indicators would now be included.
Burrage et al.^14^ 2018 Paper 2	2013	Timely implementation of WHO and ART initiation policy guidelines at country level Adoption of WHO and ART initiation policy guidelines at country level Barriers and facilitators to WHO-policy implementation	To understand the lag between guideline development and implementation, as well as the ART coverage gap, CDC assessed national HIV-guidelines and analysed Joint United Nations Programme on HIV and AIDS. Timeliness of WHO-ART guideline adoption varied by country.	Cross-sectional survey	The study analysed the levels of WHO guidelines implementation of ART initiation and how countries timeously changed and adopted country guidelines.	Angola, Botswana, Cameroon, Côte d’Ivoire, Democratic Republic of the Congo, Ethiopia, Kenya, Lesotho, Malawi, Mozambique, Namibia, Nigeria, Rwanda, South Africa, South Sudan, Swaziland, Tanzania, Uganda, Zambia and Zimbabwe	This report highlights the continuing gaps in ART coverage in PEPFAR-supported SSA countries with high HIV burden, despite expanded ART eligibility criteria. SSA countries are failing to implement WHO guidelines timeously. As of 2015, all 20 PEPFAR-supported sub-Saharan African countries included in this analysis had adopted the 2013 WHO guidelines for ART eligibility. However, adoption of the 2013 guidelines in some countries did not occur until 2 years later, that is, in 2015.	A robust data collection methodology was employed using self-reports and medical charts from both patients and healthcare workers. The limitation of the study was that it was conducted at only two treatment centres, hence the results cannot be generalised to the entire country, although we believe patients attending these two clinics are typical of those attending public health clinics throughout South Africa.
Plazy et al.^15^ 2015 Paper 3	2010 and 2013	Adoption of WHO and ART initiation policy guidelines at country level Timely implementation of WHO and ART initiation policy guidelines at country level	To describe the changes in ART initiation based on the changes on CD4 threshold changes.	Cross-sectional study.	The study aimed at describing ART initiation percentages in a large HIV programme according to the temporal changes of country ART eligibility guidelines from 2007 to 2012.	Rural KwaZulu Natal, South Africa	As temporal changes of guidelines were occurring, percentages of ART initiations significantly increased in newly ART eligible people and did not decrease in those with very low CD4 counts. It will be crucial to continue to verify the evolution of these percentages of ART initiations with future recommendations reaching near-to-universal access to ART, to ensure that individuals most in need of ART receive it on time.	The study was based on data of a large HIV treatment and care programme allowing unbiased findings and giving an accurate representation of the entire population. Some individuals may have initiated ART outside the programme, which may have led to an underestimation of the ART initiation percentages. However, this bias is likely to be limited as the Hlabisa HIV programme is decentralised in primary healthcare clinics with relatively easy access and this area is rural and poor, making it difficult for people to access ART somewhere else. Another limitation is that amongst included participants, some people may have failed to return to the clinic to receive their CD4 count result after HIV testing and thus were unaware of their status regarding ART eligibility, however we cannot provide a precise figure as this information was not collected in the database.
Hsieh et al.^6^ 2014 Paper 4	2013	Timely implementation of WHO and ART initiation policy guidelines at country level Adoption of WHO and ART initiation policy guidelines at national level Barriers and facilitators to WHO-policy implementation	The study assessed adaptation and implementation of the 2013 WHO guidelines at country level and suggests how to optimise community engagement to inform future guidelines.	Cross-sectional e-survey and e-forum discussion, FGDs	The study focused on evaluating community and HCW values and preferences on key topics to inform the development of the 2013 WHO consolidated guidelines for antiretroviral therapy in low- and middle-income countries.	Malawi and Uganda.	The findings of these community consultations have reinforced the importance of community representation, involvement, and participation in normative guidelines development. For the effective scale-up of ART programmes, it is critical to have a nuanced appreciation for the different ways in which people interact with certain services, and the role of communities and civil society in service delivery.	The data were collected from various field experts, comprising HIV clinicians, researchers, country HIV programme managers, guideline methodologists, partners from the United Nations or other development agencies and nominated representatives of civil society and/or networks of people living with HIV (selected on the basis of four criteria: technical knowledge, constituency and regional representation, previous experience with guidelines development). We ensured a balance of representation by country and sex, however, there were more females from Uganda.
Song et al.^16^ 2018 Paper 5	2015	Adoption of WHO and ART initiation policy guidelines at national level Barriers and facilitators to WHO-policy implementation	The study assessed differences in clinical benefits between individuals starting treatment at CD4 counts ≥ 500 cells/mm^3^ (early initiation) as compared with < 500 cells/mm^3^ (deferred initiation).	Observational Study	Clinical outcomes and benefits of early ART initiation at CD4 cell count 500 and below.	South Africa, Zambia, Angola, Kenya, Uganda, Lesotho and Nigeria	Mortality risk and risk for AIDS appear to be reduced amongst people living with HIV with early initiation of ART, based on current WHO guidelines, as compared with those with deferred initiation of ART (< 500 cells/mm^3^).	The study used a large sample size from all the countries and tried to identify facilities with similar structures and resources. Observational studies inherently are limited because they may provide a relatively lower quality of evidence than randomised controlled clinical trials. Furthermore, it is possible that the data density such as frequency of follow-up visits and clinical assessment between these periods may have affected our results.
Beck et al.^3^ 2006 Paper 6	2002	Timely implementation of WHO and ART initiation policy guidelines at country level Adoption of WHO and ART initiation policy guidelines at national level	The study investigated the existence of national ART guidelines in SSA-countries by the WHO and compared their content with the 2002 WHO-ART guidelines.	Observational Study	Questionnaires were sent to countries identified by WHO as requiring special attention for developing HIV-therapeutic and preventive health services, because of their high HIV-burden or because of their strategic importance in the region in terms of being able to scale-up HIV-therapeutic and preventive health services.	43 Sub-Saharan African countries	Most countries had developed national ART guidelines as part of a comprehensive national HIV programme. Concordance with WHO recommend-ations were strong on starting the first-line ART regimens and routine monitoring but weaker for second-line recommendations.	This analysis was limited to 43 WHO ‘3 by 5’ focus countries and did not involve all middle- and lower-income countries currently scaling up HIV treatment and care. This evaluation was focused on the development of national guidelines based on WHO recommendations and did not consider their effective implementation and use of the guidelines at health facility level, and substantive differences may well exist between the development of national guidelines and programme implementation resulting in actual clinical practice.
Duber et al.^17^ 2015 Paper 7	2013	Timely implementation of WHO and ART initiation policy guidelines at country level Adoption of WHO and ART initiation policy guidelines at national level Barriers and facilitators to WHO policy implementation	The study examined if WHO guidelines were adopted into practice in Kenya, Uganda and Zambia and the pace at which they were adopted at the health-facility level.	Observational analysis.	The level at which countries in regions of high HIV and AIDS burden, including Kenya, Uganda and Zambia, adopted WHO guidelines into their national guidelines	Kenya, Uganda and Zambia.	Patient-level data from a wide range of ART facilities in Kenya, Uganda and Zambia supports the assertion that national HIV programmes have moved quickly to adopt WHO-ART first-line treatment recommendations into clinical practice.	This study benefits from a large and diverse sample in terms of time, geography and facility type, but it is not without limitations. Despite efforts to sample from all patient charts, facilities use different practices in storing charts of dead or defaulted patients, and this may have differentially affected the sample of charts across facilities. Electronic data records were from a small number of facilities, whilst for the majority of facilities, chart extractions were performed by hand; therefore, it is possible that the quality of the data included differs for these facilities. However, a separate analysis of these facilities finds that they are within the expected range of prescribing patterns. Furthermore, as charts were weighted based on the size of the ART programme in the year of ART initiation, we do not expect that the facilities with electronic records had undue influence on our overall descriptive findings.
Hontelez et al.^18^ 2011 Paper 8	2010	Adoption of WHO and ART initiation policy guidelines at national level Barriers and facilitators to WHO-policy implementation	Quantifying the potential net costs and life-years saved because of the 2010 WHO guidelines compared with treating patients at ≤ 200 cells/µL.	Quantification and costing model	The study aimed at estimating the impact of fully adopting the new WHO guidelines on HIV-epidemic dynamics and associated costs.	Hlabisa sub- district of UMkhanyakude in KZN, South Africa	The findings show that starting ART at CD4 ≤ 350 recommended by WHO will lead to an increase in programme costs, but significantly more patients on ART. Compared with ART initiation at CD4 ≤ 200 initiating ART according to the new WHO guidelines will result in a cumulative net cost-saving starting around 2026.	The baseline predictions methodology concerning the Hlablisa sub-district could have been too optimistic for South Africa as a whole, where dropout rates are higher, health-seeking behaviour is less. However, the sensitivity analysis shows that these differences have a limited impact on the timing of the break-even point and the number of life-years saved. This can be explained by the fact that we compare two scenarios (ART at ≤ 200 cells/µL versus ≤ 350 cells/µL), which are both largely affected in the same way, so that the comparison between the two remains relatively unchanged.
Kuznik et al.^7^ 2016 Paper 9	2015	Timely implementation of WHO and ART initiation policy guidelines at country level. Adoption of WHO and ART initiation policy guidelines at national level Barriers and facilitators to WHO-policy implementation	The study evaluated the cost-effectiveness of immediate versus deferred ART- initiation amongst patients with CD4 cell counts exceeding 500 cells/mm^3^ in four resource-limited countries according to the 2015 WHO-ART recommendations.	Cost-effectiveness analysis	The study focused on evaluating if treatment for all patients with HIV would pose an additional strain for national ART programmes, particularly amongst those that were already struggling to meet treatment targets based on the previous CD4+ cell count threshold of 500 cells/mm^3^ proposed by the WHO.	South Africa, Nigeria and Uganda	In the studied countries, immediate versus deferred initiation of ART in HIV-positive patients with CD4+ cell counts above 500 cells/mm^3^ is cost-effective and likely cost saving. The findings support the recommendation for resource-limited countries to provide ART for all HIV-infected patients even though there were delays in policy implementation.	The 5-year Markov model used in the study allowed annual cycles to be compared including patients at different CD4 count threshold, which resulted in robust findings to changes in model parameters as observed in our one-way sensitivity analyses, and simultaneous variation in multiple parameters as observed in probabilistic sensitivity analyses. The START trial that serves as the clinical basis for our cost-effectiveness model was largely conducted outside of SSA and more than half of enrolled patients were MSM, a mode of transmission that is less commonly reported in SSA.
Ross et al.^19^ 2014 Paper 10	2013	Timely implementation of WHO and ART initiation policy guidelines at country level Adoption of WHO and ART initiation policy guidelines at national level Barriers and facilitators to WHO-policy implementation	The aim of the study was to quantify the impact of revised ART initiation thresholds on the outcome of cluster-randomised treatment-as-prevention trials, and assess how changes in trial characteristics could be used to augment the observed incidence reduction in the context of policy change.	Cost-Effectiveness and HIV- transmission analysis models	The study focused on assessing the incidence reduction using the revised (CD4 < 500 c/mm^3^) and prior (CD4 < 350 c/mm^3^) control ART initiation thresholds. In addition, it also evaluated changes to trial characteristics that could bolster the incidence reduction.	KwaZulu Natal, South Africa	Implementing the 2013 WHO HIV-treatment threshold could substantially improve the incidence reduction in HIV-population as seen in prevention trials. The feasibility of HIV-population prevention trials should be reassessed as implementation of treatment guidelines evolves.	The cross-cluster contamination method that was used proved to be highly influential on trial outcomes. The model of HIV transmission used was neither age- nor sex-stratified and did not explicitly account for behavioural factors such as concurrent sexual partnerships or sexual networks. Likewise, sex differences in HIV testing frequency and ART uptake were not modelled.
Walensky et al.^5^ 2010 Paper 11	2010	Timely implementation of WHO and ART initiation policy guidelines at country level Adoption of WHO and ART initiation policy guidelines at national level Barriers and facilitators to WHO-policy implementation	The study aimed to answer the question whether countries should begin by replacing stavudine with tenofovir or by making CD4 count monitoring universally available as recommended by the 2010 WHO guidelines. Use of a model-based analysis with data from South Africa to project the clinical and economic outcomes of alternative stepwise implementation scenarios towards the 2010 WHO and ART guidelines	Cost-effectiveness and survival analysis model	The article considers what to do first in resource-limited settings where immediate implementation of all the 2010 WHO recommendations is not feasible considering that many countries in SSA were still struggling to implement 2006 guidelines.	South Africa	In settings where immediate implementation of all the new WHO treatment guidelines is not feasible, ART initiation at CD4 < 350 cells/µL provides the greatest short- and long-term survival advantage and is highly cost-effective, however considering the high HIV-incidence and prevalence in South Africa meeting the targets timely would be a challenge. Limited infra-structure in implementing the 2010 guidelines was a challenge.	The article only focused on one aspect of mathematical models and ignored other aspects, which might influence cost implications. The study also used a simulation model combining clinical and cost data for HIV treatment into this model and then used it to project survival and costs in a hypothetical group of South African HIV-positive patients. This strengthened the findings of the study because simulation provides a unique opportunity to highly control the environment, scenario and other features that are often unpredictable and inconsistent in real-life clinical care.
Stanecki et al.^20^ 2010 Paper 12	2006 and 2010	Timely implementation of WHO and ART initiation policy guidelines at country level Adoption of WHO and ART initiation policy guidelines at country level Barriers and facilitators to WHO-policy implementation	The study estimated the number of adults (age ≥ 15 years) in need of ART from 1990 through 2009 based on the 2006 WHO guidelines and, secondly, estimated the number of adults (age ≥ 15 years) eligible for ART based on the revised 2010 WHO guidelines for the same time period in low- and middle-income countries, with a primary focus on SSA discussing the implication of these revisions	Retrospective study. Time series estimates of ART-initiation models	The ART need estimates based on ART-eligibility criteria promoted by the 2010 WHO guidelines were compared with the need estimates based on the 2006 WHO guidelines.	Botswana Cameroon Central African Republic Kenya Lesotho Malawi Mozambique South Africa Swaziland Uganda United Republic of Tanzania Zambia Zimbabwe	When adopting the new recommendations, countries failed to adapt their planning process to accelerate access to life-saving drugs to those in need. These recommendations have a significant impact on resource needs as countries in SSA struggle to implement WHO policies on time. The number of people in low- and middle-income countries eligible for ART under the revised 2006 WHO guidelines was 10.1 million compared with the estimated 14.6 million people in need under the 2010 guidelines	The study used multicountries to ensure high quality evidence from experts and multiple comparison of various national guidelines. Whilst Spectrum work well for countries where survey-derived data constitute the bulk of the surveillance data, they are not well adapted for countries that rely mostly on HIV and AIDS case reporting for HIV surveillance.
Labhardt et al.^21^ 2012 Paper 13	2006 and 2010	Timely implementation of WHO and ART initiation policy guidelines at country level Adoption of WHO and ART initiation policy guidelines at national level Barriers and facilitators to WHO policy implementation	The study compared the rate of adoption of the new guidelines and substitution of first-line drugs by health centres (HC) and hospitals in two catchment areas in rural Lesotho.	Retrospective cohort analysis	The study aimed at comparing nurse-based ART initiation at health centres in terms of adherence to treatment guidelines after the introduction of the 2006 guidelines and number of drug substitutions because of side effects.	Lesotho	Health centres took longer to adopt the new guidelines and substituted drugs less frequently because of limited knowledge on policy-change implementation Decentralised ART programmes need close support, supervision and mentoring to absorb new guidelines and to adhere to them.	It is a retrospective analysis, patients have not been randomly assigned to health centres or hospitals. This results in two cohorts with quite different baseline characteristics that may interfere with the assessed outcome variables. However, in the methodology patients were stratified according to the type of the facility where they received ART: health centres and hospitals. Analyses run, are adjusted for all baseline characteristics and the results remained significant. However, there might be other confounders that have not been assessed.
Teasdale et al.^22^ 2015 Paper 14	2006 and 2010	Timely implementation of WHO and ART initiation policy guidelines at country level Adoption of WHO and ART initiation policy guidelines at national level Barriers and facilitators to WHO policy implementation	Determine time to ART initiation amongst patients eligible at enrollment compared with those ineligible or of indeterminate eligibility who become eligible during follow-up	Retrospective study	The study examined time to ART eligibility amongst adult patients (≥ 15 years of age) and time to ART initiation amongst eligible patients receiving care at health facilities in Rwanda from 2005 to 2010 according to WHO guidelines.	Rwanda	There were higher rates of ART initiation within 3 months amongst patients who were ART eligible at enrollment. From 2006 to 2011, earlier initiation of ART after eligibility was observed likely reflecting improved programme quality. The Rwanda National HIV Care and Treatment Programme have achieved significant success in scaling up ART with 94% of eligible patients receiving treatment in 2012. Rwanda was also one of the first countries in SSA to adopt a higher CD4+ threshold for ART eligibility, instituting ART initiation at CD4+ ≤ 350 by July of 2007 proving benefits of timeous ART initiation.	The strengths of this study include the large and representative cohort; the 31 033 HIV-infected ART-naive adults included in this analysis represent 24% of all adult patients enrolled in care in Rwanda between 2005 and 2010. Patients in the analysis came from 41 different health facilities ranging in size from primary health clinics to large district hospitals and were located in both rural and urban areas. The use of routinely collected data from HIV care and treatment programmes is both an asset and limitation of this analysis. Although highly representative of actual care in Rwanda, the data do not include variables of interest, such as viral load and patient demographic characteristics that might be important predictors of ART initiation, such as distance of residence from health facility.
Konings et al.^23^ 2012 Paper 15	2010	Timely implementation of WHO and ART initiation policy guidelines at country level Adoption of WHO and ART initiation policy guidelines at country level Barriers and facilitators to WHO-policy implementation	The study assessed the implications of implementing the WHO’s 2010 guidelines for ART initiation in adults and adolescents with HIV-infection compared with the earlier threshold.	Retrospective and prospective medical chart reviews	Study estimated the total number of patients who would need ART if Ethiopia adopted the 2010 guidelines, the number of patients needing ART based on current guidelines were added to the number of asymptomatic patients enrolled in pre-ART with a CD4+ count > 200 but ≤ 350 cells/mm^3^.	Addis Ababa (Ethiopia)	Without concurrent increases in funding and governmental support, it will not be possible to scale up ART to accommodate the increased patient demand in Ethiopia. These increased costs are not currently affordable for Ethiopia, which decided to continue observing the 2006 ART guidelines. Whilst the 2010 revision is sound in principle and value, resources in Ethiopia are not enough to absorb the ensuing increased demand for existing services. Findings proved that there were shortages in staff to initiate ART because of increased numbers of eligible individuals.	Nineteen health centres were used as research sites offering a large representative sample of patients on ART in health centres in Ethiopia. Some of the medical charts had missing information affecting the quality of collecting data in some files.
Walsh et al.^24^ 2017 Paper 16	2015	Timely implementation of WHO and ART initiation policy guidelines at country level Adoption of WHO and ART initiation policy guidelines at national level Barriers and facilitators to WHO-policy implementation	The study was designed to determine the feasibility, acceptability, affordability and scalability of offering early antiretroviral treatment to all HIV-positive individuals in Swaziland’s public health system based on the WHO 2015 ART initiation guidelines	Prospective 3-year stepped-wedge randomised control study	The study measured how eligible individuals accepted immediate ART initiation, levels of drug stock out, staff preparedness on implementing UTT, retention and viral suppression patient. They also measured cost per patient per year.	Swaziland	The economic evaluation proved to be a burden on Swaziland’s public sector health system with scaling up numbers on early ART initiation. There were continuous drug shortages in most facilities, which resulted in delayed initiations.	The study was a randomised control study, which used both quantitative and qualitative methods resulting in high-impact evidence.

ART, antiretroviral therapy; CDC, Centre for Disease Control and Prevention; FGDs, focus group discussions; HCW, healthcare workers; KZN, KwaZulu Natal; SSA, Sub-Saharan Africa; UTT, universal test and treat; WHO, World Health Organization.

### Ethical consideration

Ethical approval was obtained from the University of KwaZulu-Natal Biomedical Research Ethics Committee (UKZN BREC, reference number: BREC/00000819/2019).

## Results

### Overview of selected studies

We reviewed 16 studies from an initial collection of 293 articles in Google Scholar, PubMed, Cochrane, Embase and EBSCOhost (Figure 1). We only reviewed studies that examined how different SSA countries adopted changes in WHO and ART initiation guidelines based on CD4 threshold and how the guidelines have impacted ART programmes in SSA. The following four themes were identified from the 16 papers: (1) Adoption of WHO and ART initiation policy guidelines at country level in SSA, (2) timely implementation of WHO and ART initiation policy guidelines at country level, (3) barriers and facilitators to WHO policy implementation in SSA and (4) characteristics at country level.

Of the 16 reviewed articles 4 (articles 4, 13, 14 and 16) addressed all 4 themes, 8 articles addressed 3 themes (articles 1, 2, 7, 9, 10, 11, 12 and 15) and 4 articles (articles 2, 5, 6 and 8) addressed only 2 themes. The theme of the adoption of the WHO-ART initiation guidelines at country level was dominant in all articles.

#### Theme 1: Adoption of World Health Organization antiretroviral therapy initiation policy guidelines at country level in sub-Saharan Africa

The results confirm that all the countries in SSA that are part of this review have adopted the WHO and ART initiation guidelines since 2002. Hsieh et al. reported that between July 2013 and July 2015, seven national policy documents incorporating the 2013 WHO guidelines were developed in Kenya, Malawi, Tanzania, Uganda, Zimbabwe and two in South Africa.^6^ This was further supported by Ross et al. who found that SSA countries had some national explicit policies that targeted increasing ART access in line with the WHO 2013 guidelines on ART.^19^ In his study, Hsieh et al. indicated that community consultations are crucial if policies are to be effectively implemented.^6^ Labhardt et al. found that health centres in Lesotho took longer to adopt the new guidelines because of limited knowledge of WHO policy changes.^21^

Rwanda implemented the 2006, 2010, 2013 and 2015 WHO and ART initiation guidelines in a timely manner, that is, on an average within 6 months of international release.^25^ Part of Rwanda’s success is attributed to the cooperation of government and non-governmental service providers.

#### Theme 2: The timely implementation of World Health Organization antiretroviral therapy initiation policy guidelines at country level

Teasdale et al. describe high rates of early – within 3 months – ART initiation amongst ART-eligible Rwandan patients. Indeed, by 2012, the Rwanda National HIV Care and Treatment Programme had managed to initiate 94% of eligible PLWH on ART in line with the 2006 and 2010 WHO guidelines. Rwanda was also one of the first countries in SSA to implement the higher CD4+ initiation threshold for ART eligibility.^22^ In an observational study in Kenya, Uganda and Zambia, Duber et al. indicate that national HIV programmes have implemented WHO 2013 guidelines at health facility level.^17^ These findings suggest that several countries have moved quickly to align with the WHO.

However, in a study conducted in 15 SSA countries, facilities were slow to align with the WHO’s 2006 and 2010 guidelines. They experienced delays in the actual implementation and expanding access to ART.^20^ Burrage et al. noted that few Tanzanians were initiated on ART at CD4 counts of ≤ 500/µL in 2015 despite the country’s earlier adoption of the 2013 WHO guidelines. As a result, only 64% of eligible PLWH were initiated on treatment.^14^ Stanecki et al. recorded that the number of PLWH eligible for ART in low- and middle-income countries (LMICs) under the revised 2010 WHO guidelines was 14.6 million at a time when only an estimated 10.1 million people actually received ART.^20^ As of 2015, all 20 SSA-supported U.S. President’s Emergency Plan for AIDS Relief (PEPFAR) countries had adopted the 2013 WHO guidelines for ART eligibility. Nevertheless, alignment and implementation with national guidelines took at least 2 years in all 20 countries.^14^ This demonstrates the failure of SSA countries to align and implement country guidelines timeously with the WHO.

#### Theme 3: Barriers to and facilitators of antiretroviral therapy initiation policy implementation

Fourteen studies examined the barriers to and facilitators of ART-initiation policy implementation in SSA. Ambia et al. reported a significant increase in ART initiations, from 42% to 87%, in some facilities in the urban centres of Kenya, Malawi, South Africa (SA), Tanzania, Uganda and Zimbabwe.^13^ Healthcare workers’ (HCWs) attitudes were found to be both a barrier and a facilitator of implementation at the facility level. Teasdale et al. reported that positive learning attitudes from HCWs were found to be an enabler for WHO policy adoption in Rwanda. Furthermore, the Rwandan government’s health department assembled a task team to ensure that the entire country was supported in the implementation of the revised guidelines.^22^ Hsieh et al. found, however, that HCWs in Malawi and Uganda were slow to implement the 2013 WHO guidelines because their communities ‘had not been consulted and hence lacked understanding’ of the guidelines.^6^ Similarly, Labhardt et al., in Lesotho found that HCWs especially in rural facilities, took longer to adopt and implement the 2006 and 2010 guidelines because of limited training.^21^ There was little support, mentoring and supervision and overall, less knowledge of health policy. The trainings were conducted in the cities. Travel from remote areas proved a challenge as facilities would have been left without clinical staff. The authors make the point that the government did not make sufficient effort to deploy trainers in the remote areas where more people needed the services.

The cost-effectiveness articles namely 8, 9, 10 and 11, in Table 1, indicate that economic constraints hindered various countries from implementing guidelines timeously. An Ethiopian study by Konings et al., revealed major financial constraints for the state even before ART services could be expanded as per the 2013 WHO guidelines. The government continued implementing the 2006 ART guidelines for more than a year after the 2010 guidelines were released because their financial capacity could not absorb the increased demand.^23^ Hontelez et al., in rural SA, reported that changes to the 2010 WHO guidelines led to an increase in programme costs requiring the SA government to add at least ZAR 3 billion to the healthcare budget to allow for an increase in personnel and medication.^18^

Most facilities in SSA failed to fully implement the policy guidelines on time because of limited ARV-stock.^13^ In a study from Swaziland, ARV-shortages delayed the implementation of the 2015 WHO guidelines on UTT. The available stock was not sufficient for those already on treatment.^24^ Walensky et al. reported that delays in obtaining baseline blood-test results delayed the SA-implementation of ART-guidelines in 2010. The 2-week turnaround time resulted in people not returning for results. Laboratory services were not readily accessible in rural areas and specimen-transport-delays resulted in the samples clotting and being discarded.^5^ Staff shortages in Ethiopia were identified as a barrier to implementation of the 2010 ART guidelines. In some facilities, there was neither a doctor nor a qualified nurse trained to initiate ART and PLWH had to be referred to distant hospitals.^23^

#### Theme 4: Characteristics at country level

World Health Organization guidelines are based on the best available scientific evidence and are directed to the ART-needs of LMICs. International guidelines unfortunately cannot speak to the individual economic and social realities of individual SSA countries. Of the 20 countries addressed in this review, there are nonetheless considerable similarities such as strained healthcare systems, structural and operational barriers and the need of cost-cutting measures to support healthcare systems. With the largest ART-programme on the continent, SA also carries the largest ART-related financial burden.^8^ Nigeria and Uganda have similar challenges.^7^ Funding-cuts from international donors exacerbate these challenges.^12^ Burrage et al. had noted that despite the expanded ART eligibility criteria, 20 PEPFAR-supported SSA countries with a high HIV-burden, had funding cuts before the release of the 2013 guidelines. This created continuing regional gaps in ART coverage.^14^ Drug-stock outs have been reported from Kenya, Malawi, SA, Tanzania, Uganda and Zimbabwe.^13^ Walsh et al. reported a similar challenge in Swaziland.^24^ This review has highlighted delays in aligning and implementing the WHO-ART-initiation guidelines in 20 SSA countries.^14^ This suggests a need for greater guidance with regard to strategy and implementation in the communities of SSA.

## Discussion

This review provides detailed information regarding WHO and ART initiation guidelines on CD4 count threshold changes and adoption of the guidelines in SSA. There were some variations in study designs, however, all the articles focused on CD4 ART-initiation changes in the WHO guidelines. The findings indicate that delays in adoption and implementation were frequent and widespread throughout SSA. We employed a thematic analysis and identified four crucial themes that were in all the articles. Several barriers to implementing the guidelines were identified. These include costs related to providing ART to eligible individuals, the shortage of staff and drugs in healthcare facilities and limited training of staff when guidelines were changed.

Our findings are consistent with those of Pell et al., who reported that the implementation of the 2015 guidelines took > 12 months to be adopted in all SSA countries after their official release.^1^

Mikkelsen et al. noted that in an effort to contain the demand for ART, most African countries were forced to defer treatment-initiation to those eligible PLWH who were well.^26^ Whilst policy is well intentioned, it is informed only by epidemiological data. The state of the healthcare system and sociocultural factors are critical for controlling and ending the epidemic. Our analysis of the financial, infrastructural, human resources for health and governance landscape in SSA, the feasibility associated with costs of implementing a UTT programme indicates health systems and societal perceptions related shortcomings. Although with clinical benefits, increasing the CD4 threshold has implications that reverberate across sectors: it affects budgets, infrastructure and human resources.

The WHO-ART guidelines are crafted by an international committee of experts drawn from rich and poor nations whose mandate is to provide the world’s low- and middle-income countries (LMICs) with affordable high-quality ART guidelines. Historically, ART-guideline development in high-income countries is independent of the WHO and takes a more local character, for example, the International AIDS Society (IAS)-USA division, the Southern African HIV Clinicians Society, the European AIDS Clinical Society (EACS), the British HIV Association and the ASIA-Pacific HIV Society, etc. Liaison between the WHO and these regional societies and associations is constant. WHO guidelines committee members are also members of their national HIV-agencies. International ART guidelines are almost never produced in isolation.

Local guidelines frequently predate the release of the WHO’s guidelines as local bodies require less administration/bureaucracy and can respond to new data in real time, for example, UTT and the Insight-Start and the Temprano Studies, dolutegravir in first-line ART and the ADVANCE Trial, etc.^27^ Mehraj et al. noted that Canada implemented the 2002 WHO and ART guidelines 2 months before its general release.^28^ Canada had all the required capacity with regard to resources and regular staff trainings as well as mentoring in implementing the guidelines. Within a space of 1 month after the release of the 2015 WHO and ART initiation guidelines, 60% of the facilities in Spain were already implementing rapid ART initiation.^29^ This suggests that Spain had already started preparing for the changes based on EACS guidelines. Larsen et al. revealed that despite significant funding from PEPFAR, the South African National Department of Health is still failing to implement rapid ART initiation. Indeed most SSA countries have experienced fundings cuts in the past few years.^30^

There is a worrisome trend in SSA countries concerning the national adoption of the WHO-ART initiation guidelines. This may explain why countries in SSA are still struggling to achieve the 90-90-90 target. Despite the increase in HIV testing, rapid ART initiation based on the 2015 WHO guidelines are yet to be achieved in SSA. Furthermore, there is need for African governments to seriously consider local situations and experiences when embracing global guidelines.

## Limitations

One of the limitations of our study is that we reviewed data from SSA and possibly excluded some important articles published in languages other than English. The study included only articles focusing on CD4 threshold changes on ART initiation. More articles might have been captured if language and the CD4 threshold had not been a filter.

## Conclusion

We conclude that although countries in SSA have generally adopted the WHO-ART guidelines, implementation has frequently been delayed. We noted that the changes in guidelines were fraught with many challenges like switching from treating at a CD4 count of 200 cells/mm^3^ in 2002 to rapid ART initiation in 2015 regardless of the CD4 level. Implementation has been variable across the countries of SSA because of differences in the health systems and the availability of resources. Because of the financial burden on governments, the reduction in donor funding, the rising incidence and prevalence of HIV and sometimes and the attitudes of healthcare workers, the majority of SSA countries have experienced a delay in the implementation of the guidelines. A comprehensive approach to reduce barriers whilst enhancing facilitators may improve the situation of adopting and implementing timely ART initiation guidelines.

## References

[1] Pell C, Reis R, Dlamini N, Moyer E, Vernooij E ‘Then her neighbour will not know her status’: How health providers advocate antiretroviral therapy under universal test and treat. Int Health. 2019;11(1):36–41. 10.1093/inthealth/ihy058 30137387

[2] World Health Organization Consolidated guidelines on the use of antiretroviral drugs for treating and preventing HIV infection: Recommendations for a public health approach. Geneva: World Health Organization; 2016. 27466667

[3] Beck EJ, Vitoria M, Mandalia S, Crowley S, Gilks CF, Souteyrand Y National adult antiretroviral therapy guidelines in resource-limited countries: Concordance with 2003 WHO guidelines? AIDS (London, England). 2006;20(11):1497–1502. 10.1097/01.aids.0000237365.18747.13 16847404

[4] WHO Guidelines Approved by the Guidelines Review Committee WHO recommendations on the diagnosis of HIV infection in infants and children. Geneva: World Health Organization; 2010. 23741779

[5] Walensky RP, Wood R, Ciaranello AL, et al Scaling up the 2010 World Health Organization HIV treatment guidelines in resource-limited settings: A model-based analysis. PLoS Med. 2010;7(12):e1000382 10.1371/journal.pmed.1000382 21209794 PMC3014084

[6] Hsieh AC, Mburu G, Garner AB, et al Community and service provider views to inform the 2013 WHO consolidated antiretroviral guidelines: Key findings and lessons learnt. AIDS (London, England). 2014;28(Suppl 2):S205–S216. 10.1097/QAD.0000000000000251 24849480

[7] Kuznik A, Iliyasu G, Habib AG, Musa BM, Kambugu A, Lamorde M Initiation of antiretroviral therapy based on the 2015 WHO guidelines. AIDS (London, England). 2016;30(18):2865–2873. 10.1097/QAD.0000000000001251 27662547

[8] Danel C, Moh R, Gabillard D, et al A trial of early antiretrovirals and isoniazid preventive therapy in Africa. N Engl J Med. 2015;373(9):808–822. 10.1056/NEJMoa1507198 26193126

[9] Lundgren JD, Babiker AG, Gordin F, et al Initiation of antiretroviral therapy in early asymptomatic HIV infection. N Engl J Med. 2015;373(9):795–807. 10.1056/NEJMoa1506816 26192873 PMC4569751

[10] Perriat D, Balzer L, Hayes R, et al Comparative assessment of five trials of universal HIV testing and treatment in sub-Saharan Africa. J Int AIDS Soc. 2018;21(1):e25048 10.1002/jia2.25048 PMC5810333 29314658

[11] Nash D, Yotebieng M, Sohn AH Treating all people living with HIV in sub-Saharan Africa: A new era calling for new approaches. J Virus Eradic. 2018;4(Suppl 2):1–4. 10.1016/S2055-6640(20)30340-X PMC6248848 30515307

[12] Moher D, Liberati A, Tetzlaff J, Altman DG, PRISMA Group Preferred reporting items for systematic reviews and meta-analyses: The PRISMA statement. Ann Intern Med. 2009;151(4):264–269. 10.7326/0003-4819-151-4-200908180-00135 19622511

[13] Ambia J, Renju J, Wringe A, et al From policy to practice: Exploring the implementation of antiretroviral therapy access and retention policies between 2013 and 2016 in six sub-Saharan African countries. BMC Health Serv Res. 2017;17(1):758 10.1186/s12913-017-2678-1 29162065 PMC5698969

[14] Burrage A, Patel M, Mirkovic K, et al Trends in antiretroviral therapy eligibility and coverage amongst children aged < 15 years with HIV infection – 20 PEPFAR-supported sub-Saharan African countries, 2012–2016. MMWR Morb Mortal Wkly Rep. 2018;67(19):552–555. 10.15585/mmwr.mm6719a4 29771871 PMC6048945

[15] Plazy M, Dabis F, Naidu K, Orne-Gliemann J, Barnighausen T, Dray-Spira R Change of treatment guidelines and evolution of ART initiation in rural South Africa: Data of a large HIV care and treatment programme. BMC Infect Dis. 2015;15:452 10.1186/s12879-015-1207-2 26497054 PMC4620741

[16] Song A, Liu X, Huang X, et al From CD4-based initiation to treating all HIV-infected adults immediately. Front Immunol. 2018;9:212 10.3389/fimmu.2018.00212 29487595 PMC5816781

[17] Duber HC, Dansereau E, Masters SH, et al Uptake of WHO recommendations for first-line antiretroviral therapy in Kenya, Uganda, and Zambia. PLoS One. 2015;10(3):e0120350 10.1371/journal.pone.0120350 25807553 PMC4373941

[18] Hontelez JA, De Vlas SJ, Tanser F, et al The impact of the new WHO antiretroviral treatment guidelines on HIV epidemic dynamics and cost in South Africa. PLoS One. 2011;6(7):e21919 10.1371/journal.pone.0021919 21799755 PMC3140490

[19] Ross E, Tanser F, Pei P, et al The impact of the 2013 WHO antiretroviral therapy guidelines on the feasibility of HIV population prevention trials. HIV Clin Trials. 2014;15(5):185–198. 10.1310/hct1505-185 25350957 PMC4212337

[20] Stanecki K, Daher J, Stover J, Beusenberg M, Souteyrand Y, Garcia Calleja JM Antiretroviral therapy needs: The effect of changing global guidelines. Sex Transm Infect. 2010;86(Suppl 2):ii62–ii66. 10.1136/sti.2010.046177 21106517 PMC3173806

[21] Labhardt ND, Sello M, Lejone T, et al Adoption of new HIV treatment guidelines and drug substitutions within first-line as a measure of quality of care in rural Lesotho: Health centers and hospitals compared. Trop Med Int Health. 2012;17(10):1245–1254. 10.1111/j.1365-3156.2012.03051.x 22845835

[22] Teasdale CA, Wang C, Francois U, et al Time to initiation of antiretroviral therapy amongst patients who Are ART eligible in Rwanda: Improvement over time. J Acquir Immun Defic Syndr. 2015;68(3):314–321. 10.1097/QAI.0000000000000432 PMC5074335 25415291

[23] Konings E, Ambaw Y, Dilley K, Gichangi P, Arega T, Crandall B Implications of adopting new WHO guidelines for antiretroviral therapy initiation in Ethiopia. Bull World Health Organ. 2012;90(9):659–663. 10.2471/BLT.11.089599 22984310 PMC3442386

[24] Walsh F, Bärnighausen T, Delva W, et al Impact of early initiation versus national standard of care of antiretroviral therapy in Swaziland’s public sector health system: Study protocol for a stepped-wedge randomized trial. Trials. 2017;18(1):383 10.1186/s13063-017-2128-8 28821264 PMC5563033

[25] Mutimura E, Addison D, Anastos K, et al Trends in and correlates of CD4+ cell count at antiretroviral therapy initiation after changes in national ART guidelines in Rwanda. AIDS (London, England). 2015;29(1):67–76. 10.1097/QAD.0000000000000520 PMC4487360 25562492

[26] Mikkelsen E, Hontelez JAC, Nonvignon J, et al The costs of HIV treatment and care in Ghana. AIDS (London, England). 2017;31(16):2279–2286. 10.1097/QAD.0000000000001612 PMC5642329 28991025

[27] Venter WDF, Moorhouse M, Sokhela S, et al Dolutegravir plus two different prodrugs of tenofovir to treat HIV. N Engl J Med. 2019;381(9):803–815. 10.1056/NEJMoa1902824 31339677

[28] Mehraj V, Cox J, Lebouché B, et al Socio-economic status and time trends associated with early ART initiation following primary HIV infection in Montreal, Canada: 1996 to 2015. J Int AIDS Soc. 2018;21(2):1-N.PAG 10.1002/jia2.25034 PMC5804015 29412520

[29] Suarez-Garcia I, Gonzalez J, Berenguer J, et al Reasons for noncompliance with the national guidelines for initial antiretroviral therapy of HIV-infected patients in Spain, 2010–2015. Enfermed Infecc Microbiol Clin. 2019;37(9):580–587. 10.1016/j.eimc.2019.02.007 30982676

[30] Larsen A, Cheyip M, Tesfay A, et al Timing and predictors of initiation on antiretroviral therapy amongst newly-diagnosed HIV-infected persons in South Africa. AIDS Behav. 2019;23(2):375–385. 10.1007/s10461-018-2222-2 30008050 PMC6331268

